# Mini Review: Highlight of Recent Advances and Applications of MALDI Mass Spectrometry Imaging in 2024

**DOI:** 10.1002/ansa.70016

**Published:** 2025-05-10

**Authors:** Yuen Tung Ngai, Darren Lau, Parul Mittal, Peter Hoffmann

**Affiliations:** ^1^ UniSA Clinical and Health Sciences, Health and Biomedical Innovation University of South Australia Adelaide South Australia Australia

## Abstract

Matrix‐assisted laser desorption/ionisation mass spectrometry imaging (MALDI‐MSI) is an emerging imaging tool that allows visualisation of hundreds of analytes unbiasedly in a single experiment. This paper highlights the adaptations of MALDI‐MSI in different context in 2024, such as clinical diagnostic, pharmacology, forensics applications, plant metabolism and biology. Challenges and advancements were also discussed regarding sample preparation, instrumentations, data analysis, and integration of machine learning in the trend of single cell resolution and multi‐omics. There are still rooms for improvements in sensitivity, spatial resolution, acquisition algorithm and data integration across multi‐omics data to enable MALDI‐MSI at subcellular level.

## 1 Introduction

Since the development of mass spectrometry (MS) back in the early 20th century, it has expanded into various specialised techniques, including mass spectrometry imaging (MSI), which enables the visualisation of molecular distributions across biological samples [1]. As compared to conventional MS, such as liquid chromatography tandem mass spectrometry (LC‐MS/MS), which analyses the whole sample in‐solution as a single entity, MSI collects mass spectra within a virtual equidistant pixel, from 5 to 150 µm^2^, across a thin tissue section. Data is acquired as individual spectrum per pixel, which is then linked to specific locations, allowing the spatial mapping of analytes. This technology allows multiple analytes to be detected in the same experiment in both targeted and untargeted approaches. Therefore, it has emerged as a promising technology for discovery studies aimed at determining the localisation of analytes in an unbiased approach. Herein, we discuss the challenges and advancements of MALDI‐MSI technology in 2024, focusing on sample preparation, instrumentation, data acquisition and analysis. The implementation of MALDI‐MSI for diverse applications and its potential in spatial multi‐omics are also described in this mini‐review.

### 1.1 History

MSI was first conceptualised more than 50 years ago, when MS was combined with secondary ion mass spectrometry (SIMS), a surface analysis technique where ions were sputtered from a sample's surface and then analysed via MS [2]. While it was initially used for analysing semiconductor surfaces, MSI started to gain widespread attention in the biomedical field when Caprioli [3] and Spengler [4] demonstrated the use of MALDI‐MSI for the analysis of proteins and peptides. Other ionisation techniques were also developed, such as desorption electrospray ionisation (DESI) with less complex sample preparation procedures [5], and laser ablation inductively coupled plasma (LA‐ICP) for elemental analysis [6].

**FIGURE 1:**
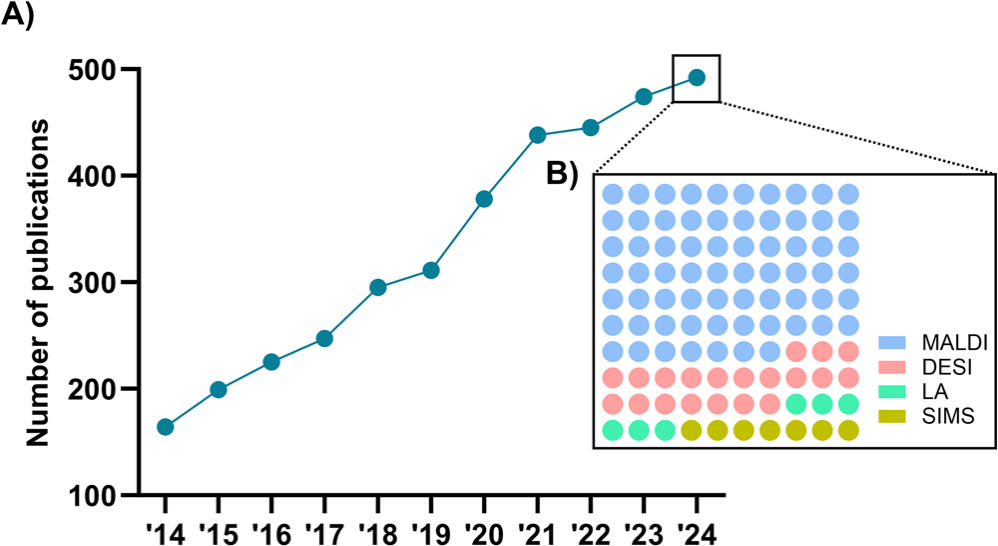
Publication trends of MSI studies. (A) Line graph showing the number of PubMed‐listed publications with search query of ‘mass spectrometry imaging’ from year 2014 to 2024. (B) Proportional representation of four ionisation sources mentioned in MSI studies published in 2024. Search queries used were ‘mass spectrometry imaging’ AND ‘MALDI’ OR ‘SIMS’ OR ‘DESI’ OR ‘laser ablation’. Data was obtained as of 31 December 2024.

There is a linear increase in MSI studies from 2010 to 2024 (Figure 1A). As a soft ionisation technique that covers a wide mass range, MALDI allows the detection of a broad spectrum of compounds from small metabolites to large proteins [7], thus becoming one of the most commonly employed ion sources (Figure 1B). The recent trajectory of advances to improve sensitivity, resolution and throughput in MALDI‐MSI, especially in 2024, continues to expand into more applications, which will be the centre of interest in this mini‐review.

### 1.2 Working Principles of MALDI‐MSI

**FIGURE 2:**
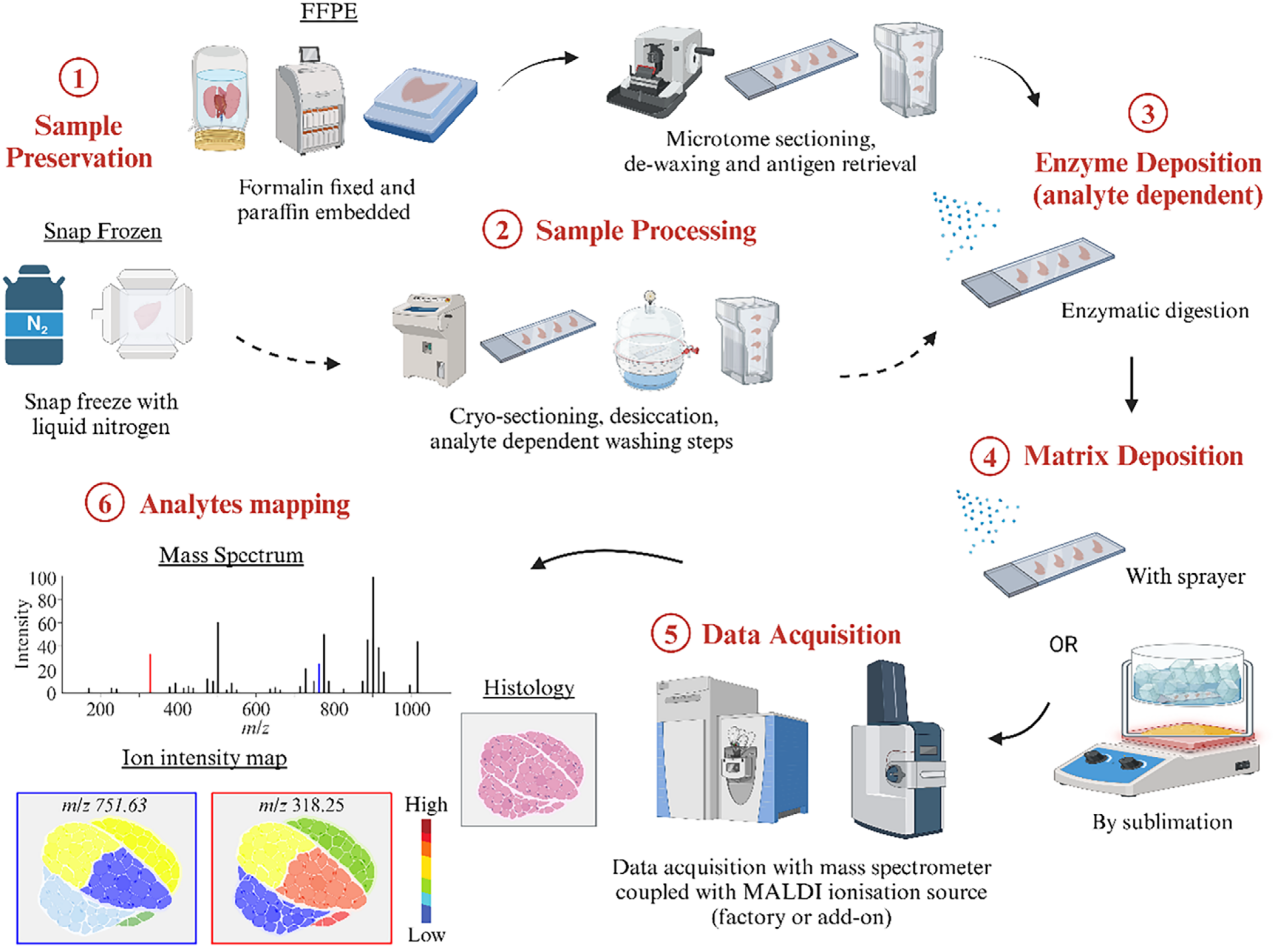
A typical MALDI‐MSI workflow. Key steps include: (1) Tissue preservation via formalin‐fixing or snap‐freezing and sample embedding. (2) Embedded tissues are sectioned and mounted on ITO slides. Sample processing steps such as de‐waxing, antigen retrieval and washing may follow based on analyte(s) of interest. (3) Samples may undergo enzymatic digestion. For example, trypsin and PNGase F are applied for the analysis of peptides and *N‐*glycans respectively. (4) Matrix is deposited onto the tissue by spraying or sublimation. (5) Data is acquired in a MS coupled with MALDI source. (6) Generation of ion distribution images by mapping ion intensities obtained from the MS to specific locations in the tissue, creating spatial maps of analyte distribution. Figure is created using the BioRender software.

An overview of MALDI‐MSI workflow is shown in Figure 2. The general MALDI‐MSI workflow for tissue analysis begins with sample preservation. Depending on the analytes intended for detection, the most commonly used methods are formalin fixation for microtome sectioning, or snap freezing using liquid nitrogen to preserve fresh tissues for cryo‐sectioning. The choices of embedding materials are usually paraffin for formalin fixed (FFPE) tissue and carboxymethylcellulose (CMC) or gelatin for snap frozen samples, depending on the tissue type and hardness. Despite its prominent role in traditional histology experiments, the use of optimal cutting temperature (OCT) media as the embedding material for MALDI‐MSI is forewarned due to its contribution to ion suppression, thus reducing sensitivity of analyte detection [8, 9]. After embedding and sectioning, tissue sections are mounted onto glass slides using water bath mounting for microtome‐sectioned tissue and thaw‐mounting or double‐sided adhesive tape for cryo‐sectioned tissues. Indium tin oxide (ITO) glass slides were recommended for tissue mounting so that MSI and light microscopy can be performed on the same slide [10].

Once mounted, FFPE tissues are deparaffinised and dehydrated using xylene and ethanol gradient baths, followed by antigen retrieval and enzymatic cleavage. In contrast, fresh frozen tissues undergo desiccation, washing steps, quick fixations, or enzymatic digestion, which may involve reagents such as ethanol and Carnoy's fluid (a mixture of 60% ethanol, 30% chloroform and 10% acetic acid v/v/v) [11]. Typically, trypsin is used for peptide MALDI‐MSI and PNGase F for *N*‐glycan MALDI‐MSI [12]. While both FFPE and snap frozen tissues are capable for peptide and *N*‐glycan MALDI‐MSI, the snap frozen sample requires extra washing steps to remove lipids as their ionisation efficiency is much greater than peptides and *N*‐glycans, leading to signal suppression of the analytes. Although FFPE tissues have been used for lipid analysis, snap frozen samples are still preferred to avoid the loss of lipid analytes during de‐waxing steps [13]. Matrix is then applied to the mounted sections, often using sprayer or sublimation. Where desirable, sublimation is the first choice as this solvent‐free technique minimises the diffusion of solutes across tissue regions, resulting in spatial delocalisation [14]. The matrix serves as a critical compound to promote the desorption and ionisation of the analytes of interest in MALDI‐MSI [15]. The matrix‐coated slides are then loaded into a MS, where a laser fires the matrix with analytes extracted from the tissue. The laser energy is absorbed by the matrix and analytes in the tissue, facilitating ionisation. These ionised molecules are then analysed based on their mass‐to‐charge (*m*/*z*) ratio. Mass spectra are acquired at each location as the laser fires at different spots across the tissue surface, creating a spatial map of ion intensities, which is processed into molecular distribution images that reflect the tissue's composition, using compatible data analysis software and algorithm.

## 2 Applications of MALDI‐MSI in 2024

MALDI‐MSI is increasingly being applied across various fields, transforming the way spatially resolved molecular information is utilised. This section highlights the key areas where MALDI‐MSI has made a significant impact over the past year.

### 2.1 Biomarker Discovery and Disease Pathology

**FIGURE 3:**
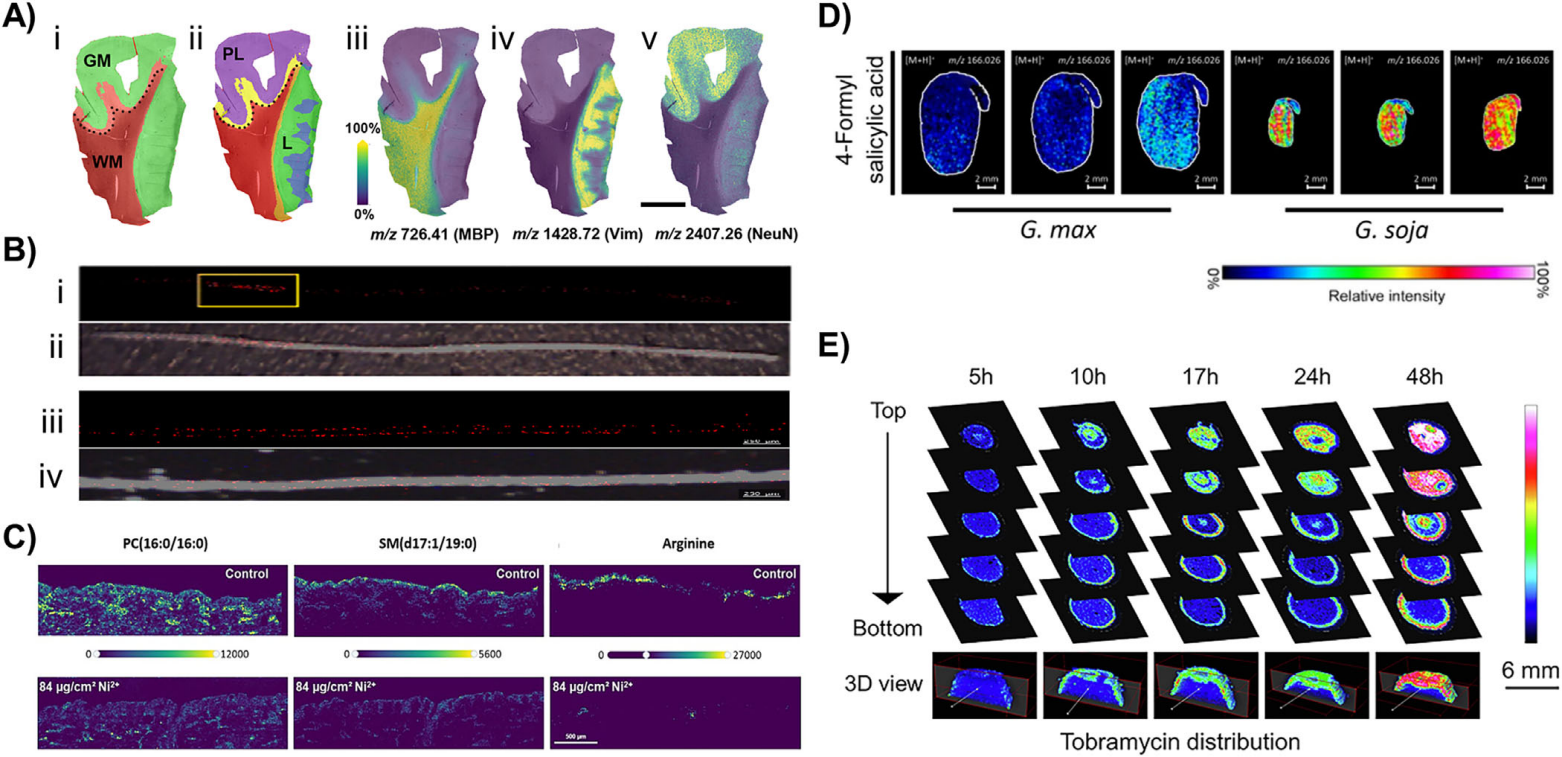
Diverse range of MALDI‐MSI applications. (A) Peptide MALDI‐MSI of focal cortical dysplasia section. (i, ii) Spatial segmentation of grey matter (GM), white matter (WM), perilesional (PL) and lesional (L) regions. (iii–v) Ion distribution images of peptides from myelin basic protein (MBP, *m*/*z* 726.41), vimentin (Vim, *m*/*z* 1428.72) and neuronal nuclei (NeuN, *m*/*z* 2407.26). Adapted and modified from Cagnoli et al. [20] (fig. 3) with permission from John Wiley and Sons. (B) (i) Ion distribution image of zolpidem ions (*m*/*z* 308.17578, indicated by red signals) and (ii) overlaid optical image of a hair sample from an individual who ingested the drug. (iii) Ion distribution image of zolpidem, and (iv) overlaid optical image of soaked hair. Adapted and modified from Ji et al. [22] (figs. S1 and S2) with permission from Elsevier. (C) Ion distribution images of a phosphatidylcholine (PC (32:0), *m*/*z* 734.56), sphingolipid (SM(d17:1/19:0), *m*/*z* 731.60) and arginine (*m*/*z* 175.11) in control and nickel‐treated skin samples. Adapted and modified from Rezaei et al. [26] (fig. 4) with permission from John Wiley and Sons. (D) Ion distribution images of 4‐formylsalicylic acid (*m*/*z* 166.026) in cultivated (*G. max*) and wild soybeans (*G. soja*). Adapted and modified from Yin et al. [39] (fig. 5) with permission from Elsevier. (E) Ion distribution images of tobramycin (*m*/*z* 468.26) in *P. aeruginosa* biofilm colonies at multiple post‐treatment stages with a three‐dimensional perspective. Adapted and modified from Shen et al. [41] (fig. 6) with permission from Elsevier.

MALDI‐MSI is extensively applied in oncology due to its ability to provide insights of tumour heterogeneity and microenvironments. Using MALDI‐MSI, researchers identified extracellular matrix (ECM) collagen peptides to differentiate non‐invasive ductal carcinoma in situ from invasive breast cancer, highlighting potential biomarkers to characterise breast cancers [16]. *N*‐glycans and proteins alterations in the ECM were also proposed as predictors of prostate cancer progression following postprostatectomy [17]. Furthermore, MALDI‐MSI has become a widely used technique in neurological research due to its ability to unbiasedly map lipid, metabolite, glycan and protein changes in brain tissues spatially. Notable research highlights from 2024 include the identification of ganglioside accumulation in amyloid beta plaque in Alzheimer's disease [18], the identification of lipid alterations linked to schizophrenia [19] and the demonstration of MALDI‐MSI's capability to differentiate lesional from perilesional regions in epilepsy using post‐surgery fixed tissue (Figure 3A) [20].

### 2.2 Spatial Pharmacology

Investigating drug delivery, penetration and accumulation is critical in pharmacokinetics, particularly for characterising absorption, distribution, metabolism and excretion (enabling targeted delivery and understanding temporal concentration changes. In 2024, several MALDI‐MSI studies were performed for this purpose on different sample types.

By using MALDI‐MSI, Tenebro and colleagues reported the accumulation of rotenone (*m*/*z* 395.1495, [M + H]^+^), a bioactive compound in plants with anti‐cancer properties, in the renal cortex region of rat kidneys after 24 h of drug administration. The authors also noted the detection of an ion with the same *m*/*z* in the control samples. However, this ion displayed a different ion mobility separation as compared to those observed in drug‐administered kidneys and the rotenone standard, highlighting the importance of ion mobility in drug characterisation [21].

In another study, researchers observed the distribution of the hypnotic drug zolpidem in the middle of a single hair shaft when the drug was ingested. In contrast, when the hair was deliberately contaminated, the drug could only be observed in the outer layers (Figure 3B) [22]. These results suggests that MALDI‐MSI can be a useful tool in a forensic context to distinguish drug ingestion and potential exogenous contamination.

### 2.3 Applications in Dermatology

The use of MALDI‐MSI in skin models is a relatively new but rapidly growing area of research. Berberine, a plant‐derived alkaloid, was shown to permeate epidermis and dermis layers via transdermal delivery using microneedle arrays [23], implying the usefulness of MALDI‐MSI in evaluating drug penetration, but using ex vivo skin models. Another study examined the accumulation of a protein kinase inhibitor, selumetinib and its degradation product in skin appendages, which may provide insights in drug formulation strategies for prolonged efficacy [24]. MALDI‐MSI was also used to investigate whether benzalkonium chloride, an antiseptic used in antimicrobial products can penetrate the human skin [25].

In addition to analysing targeted compounds, MALDI‐MSI can also be used as a spatial omics approach to investigate global biological changes. For instance, researchers have identified differential regulation of lipids or metabolites in response to nickel exposure (Figure 3C) [26], or in hypertrophic scars [27], compared to normal tissue. These findings may guide further research aimed at developing relevant treatments or medications.

### 2.4 Plant Metabolism

MALDI‐MSI has also been widely adapted in plant research, ranging from the simple purpose of visualising accumulation of bioactive compounds or metabolites [28, 29, 30] to understanding the physiology of plant growth, development or responses to stress [31, 32, 33]. MALDI‐MSI workflows on plants are often complicated by poor tissue integrity [34], but recent innovative solutions have emerged to overcome these challenges. one such solution is a novel electromagnetic field‐assisted frozen tissue planarisation technique, designed to address irregular morphology of plant tissues [35]. Excitingly, a new approach introduced last year, called RhizoMAP, allows for the study of interactions between plant roots and the surrounding soil in the rhizosphere without disrupting the root–soil organisation [36].

One of the most significant MALDI‐MSI breakthroughs in 2024 is the use of MALDI‐2 (post ionisation) in plant research. In this study, the authors revealed distinct localisation of bioactive metabolites across different leaf compartments, such as mesophyll, epidermis and vascular bundle layers [37]. Understanding the distribution of bioactive compounds in plants can then be translated into medicinal research. This translational capability is further highlighted in a study published in the same year, where ethyl caffeate and homoorientin were found to localise in the exocarp of the primary ginseng root [38]. The two compounds were tested for their pro‐inflammatory effects in vitro in the same study, suggesting that the removal of the brownish yellow exocarp can reduce side effects after ginseng consumption [38].

MALDI‐MSI is also useful in deciphering molecular mechanisms related to plant defence, stress response or growth and development. In an attempt to characterise wild versus cultivated soybeans, researchers uncovered the downregulation of metabolites related to stress tolerance in cultivated seeds (Figure 3D) [39]. These findings indicate the loss of environmental adaptability in soybeans throughout the domestication process, hence crossbreeding with wild soybeans may re‐establish stronger ecological traits in cultivated varieties. Detection of phytohormones using MALDI‐MSI also enabled the investigation of plant growth regulation during calyx abscission in Korla fragrant pear, a process critical in determining fruit quality [40].

### 2.5 Applications in Other Organisms

Apart from animals and plants, MALDI‐MSI has been applied in the field of other kingdoms over the past decade. The year 2024 was when we witnessed the efforts of researchers to push the boundaries further in this area.

Most notably, a non‐embedding moisture‐assisted cryo‐section (MACS) workflow was established for three‐dimensional (3D) metabolite MALDI‐MSI of microorganism colonies in biofilms [41]. The method was tested in both *Pseudomonas aeruginosa* and *Staphylococcus aureus* biofilms, underlining its versatility. The distribution of tobramycin antibiotic was tracked in the biofilm colonies, which revealed a non‐diffusive localisation pattern in the outer layers (Figure 3E). It was suggested that since tobramycin requires an active electron transport chain to be transported into a bacteria, the cells within the biofilm are exhibiting intrinsic resistance due to dormancy [41].

A new sample preparation step was also introduced to overcome the limitation on imaging bacterial colonies with heterogenous surfaces [42]. This workflow involves the culturing of microorganisms indirectly on an agar medium, having a polymeric membrane in between. This membrane can then be removed along with the colonies, leaving bacterial metabolites that have been released through the membrane onto the agar. MALDI‐MSI can be performed on the excised agar medium with flat surface, thus improving sensitivity and image quality [42]. Other interesting MALDI‐MSI studies include comparing metabolite abundance in wild and cultured *Trichodesmium* cyanobacterium [43], identifying glycosphingolipid changes during the maturation of liver fluke parasites (*Fasciola hepatica*) [44] and tracking Symbiodiniaceae dinoflagellates in reef‐building corals [45].

## 3 Recent Advances: Towards Spatial Multi‐Omics at Single‐Cell Resolution

MALDI‐MSI is an emerging technology that has made significant progress over the years, as underlined by the gradually increasing number of related publications. Nevertheless, there are still existing challenges and limitations associated with this technology. Researchers strive to detect as many desirable analytes as possible with high sensitivity and rich information content while achieving the highest spatial resolution. Unlike LC‐MS/MS, typical untargeted MALDI‐MSI data acquisition do not involve MS/MS, which fragments analytes for structural mapping to gain confident identifications. Consequently, obtaining a complex and informative MS spectrum is crucial in standalone MALDI‐MSI experiments. However, MALDI‐MSI often suffers from low sensitivity at high spatial resolution as the number of analytes ablated in the small sampling area by the laser determines the sensitivity. In this section, we highlighted several major advancements in 2024 that aimed to improve MALDI‐MSI experiments.

### 3.1 Sample Preparation

Sample preparation is a critical step in MALDI‐MSI experiments, ultimately influencing throughput, sensitivity, resolution and image quality. Hence, innovative techniques have been constantly developed in this initial stage of a MALDI‐MSI workflow.

#### 3.1.1 On‐Slide Derivatisation

Derivatisation enhances the stability and detectability of analytes, particularly *N*‐glycans. However, on‐tissue derivatisation has always been challenging for MSI analysis due to the inability to perform sample clean‐up. The presence of other molecules on the tissue can cause competition for ionisation, leading to ionisation suppression and reduced sensitivity for certain analytes.

In 2024, studies introduced new approaches for on‐tissue derivatisation using FFPE tissue that are compatible with MSI analysis. For instance, Cumin et al. [46] enhanced a previous protocol by utilising PyAOP (7‐Azabenzotriazol‐1‐yloxy)tripyrrolidinophosphonium hexafluorophosphate) and methylamine as reactants for the stable amidation of sialylated glycans [47, 48]. Their study showed an increase in the intensity of sialylated species at higher mass, while the intensity of *m*/*z* 1663.5 (Hex5HexNAc4) decreased following derivatisation using their 15‐min method. This method is also compatible with near‐single‐cell resolution, delivering a robust and reliable method for *N*‐glycan derivatisation in MALDI MSI [46]. The results suggest that the peak intensity of neutral *N‐*glycans may be influenced by the loss of sialylated species, emphasising the importance of derivatisation in MSI experiments involving *N‐*glycans.

#### 3.1.2 Matrix Application

The choice of matrix is crucial for the success of MALDI‐MSI experiments, as it influences polarity matching between the matrix and analytes, solvent choice, chemical stability during lengthy imaging runs and background interference. The most commonly used matrix includes CHCA (α‐Cyano‐4‐hydroxycinnamic acid), DHB (2,5‐dihydroxybenzoic acid), DHAP (2,6‐Dihydroxyacetophenone) for glycopeptides and phosphopeptides, or 9‐AA (9‐aminoacridine), SA (sinapinic acid) and DAN (1,5‐diaminonaphthelene) for lipid, peptide, glycan and protein MSI analysis. However, these matrices are not suitable for metabolites MSI, due to the interference from matrix ions, which suppress the detection of low molecular weight analytes [49].

In 2024, new matrices have been introduced to improve the sensitivity for different analytes. Bao et al. [49] demonstrated the use of 2‐hydroxy‐5‐nitro‐3‐(trifluoromethyl)pyridine (HNTP) as a matrix for metabolite MSI in the *m*/*z* range of 60–1500 successfully detecting 152 metabolites in rat brain tissues at 150 µm resolution. This matrix detected 31 more analytes than DHB [49]. Although the spatial resolution was not at single‐cell level, no issues with delocalisation or lower resolution were reported. In addition, Chen et al. [50] tested 2,4‐dihydroxy‐5‐nitrobenzoic acid (DHNBA), which outperformed DHB in detecting phytohormones in plant tissues [51, 52]. Five isoprenoid cytokinins (tZ, DHZ, ABA, IAA and ACC) were visualised with MALDI‐MSI for the first time, highlighting the importance of matrix selection in improving analyte detection [50]. Furthermore, Liang et al. [53] showed that nitro indole derivatives serve as a superior matrix in MALDI‐MSI, demonstrating higher sensitivity compared to the routinely used matrices (DHB, CHCA, SA, DAN and 9‐AA) for lipid, peptide, protein, glycan and PFOS MSI in both positive and negative ionisation mode [53]. These findings highlight the critical importance of matrix selection in enhancing the sensitivity and accuracy of MALDI‐MSI.

Crystal size plays a crucial role in spatial resolution, making it an important consideration in MALDI‐MSI experiments. In 2024, significant advancements in matrix crystallisation and deposition methods contributed to improved resolution. Spray‐based techniques involving the use of organic solvents typically produce crystals larger than 10 µm in size [54]. Alternatively, sublimation‐based methods have been developed where the solid phase matrix turns into gas under heat and vacuum, which then coats the slide cooled beneath a cold chamber [54]. This method was first successfully applied on a MALDI‐MSI study back in 2007 [14]. Sublimation‐based method creates finer crystals and prevents the analyte from re‐solubilisation and lateral migration [54].

Research in 2024 explored ways to optimise matrix performance, such as fine‐tuning crystallisation temperatures to reduce crystal size [54]. In addition, the use of conjugate polymer anchors has been investigated to minimise matrix volatilisation under high vacuum [55]. A notable achievement in 2024 was the development of a method that achieved 1.5‐µm resolution for single‐cell MSI by using an ultra‐fine pneumatic spraying system in atmospheric‐pressure scanning microprobe MALDI‐MSI (AP‐SMALDI MSI) [56]. This advancement holds promise for further improving the spatial resolution in MALDI‐MSI experiments.

#### 3.1.3 Tissue Expansion Approach

Expansion microscopy was first invented in 2015 by Chen, Tillberg and Boyden for super‐resolution microscopy with diffraction‐limited microscopes by physically expanding and magnifying fixed cells and tissues using a swellable polyelectrolyte gel [57]. In 2024, tissue expansion protocols compatible with MALDI‐MSI have been investigated. During expansion microscopy, samples are expanded isotropically, allowing more detailed observation of tissue microstructures. To enable MALDI‐MSI adaptation, the protocol has been modified to cater for cryo‐sectioning and shrinkage prevention under vacuum conditions using sodium hyaluronate polysaccharide as structural support. A two‐fold expansion was achieved via this technique to enhance morphology details [58], which further tolerates MALDI‐MSI data acquisition at single‐cell or even subcellular level.

### 3.2 Spatial Multi‐Omics

Spatial multi‐omics involves precise co‐localisation of information spatially resolved at multiple molecular layers, such as the transcriptome, proteome, glycome and metabolome, providing holistic insights into a complex biological system.

#### 3.2.1 Coupling MALDI‐MSI With Laser Capture Microdissection

Laser capture microdissection (LCM) enables researchers to selectively excise tissue regions for downstream analyses. These regions of interest are often defined using immunohistochemistry staining, which can only detect few targeted analytes due to limited availability of the antibodies, hence restricting multiplex capabilities. On the other hand, MALDI‐MSI offers a comprehensive view of the molecular landscape via unbiased mapping of the regions of interest. However, the ITO slides commonly used in MALDI‐MSI are incompatible with LCM, which requires polyethylene naphthalate (PEN) membrane slides. As a result, these analyses must be performed on separate tissue sections rather than a single section. While this approach is acceptable for most sample types, it presents significant challenges for tissues such as inflated lung, where the air‐filled spaces and irregular 3D structures can lead to substantial differences even between consecutive sections [12]. This limitation makes the workflow unsuitable for single‐cell spatial multi‐omics, where consecutive sections could not be acquired.

In 2021, Mezger et al. [59] attempted to perform LCM directly on ITO slides, but the approach proved less favourable for protein identification [59]. To overcome this limitation, Truong et al. evaluated the feasibility of using PEN membrane slides for MALDI‐MSI instead of traditional ITO conductive slides, as non‐conductive slides are compatible with the timsTOF fleX (Bruker Daltonics, Germany) system [60]. Their results showed that the relative intensity of analytes is comparable for both slide types. In addition, hierarchical clustering of spatially resolved MS data produced nearly identical segmentation maps regardless of slide type and aligned with histology information on the consecutive H&E‐stained section. The authors also proposed an innovative spatial multi‐omics approach, where lipidomic MALDI‐MSI is first used to define regions of interest for LCM mapping, followed by LC‐MS/MS proteomics, enabling a refined correlation between the two omics datasets. The MSI data, or SCiLS Lab landscape, is then exported to the Bruker region mapper software for precise mapping of regions of interest. The information is later transferred to the LCM microscope coordination system for precise LCM [60]. With the improved capability of LCM to isolate single cells and even organelles, this approach marks a significant step forward towards subcellular spatial multi‐omics.

Recent advancements in technology have significantly shifted from traditional bulk tissue analysis to the ability to examine cellular heterogeneity across tissues, offering a comprehensive view of molecular features at the level of individual cell types [61]. This progress is exemplified by the development of Deep Visual Proteomics (DVP), a pioneering approach coined by Mund et al. [62] in 2022, that enables spatial single‐cell‐type proteome analysis. By combining high‐resolution imaging, AI‐based single‐cell phenotyping, automated laser microdissection and ultrasensitive MS, DVP provides a powerful tool for dissecting the proteomic landscape of tissues at an unprecedented level of detail [63]. Given DVP's ability to provide a detailed proteomic profile of individual cell types, in 2024, Zheng et al. [64] demonstrated its accuracy and robustness in a composite case of classical Hodgkin lymphoma (cHL) and small lymphocytic lymphoma (SLL) in a single patient. Their findings revealed distinct proteome profiles in cHL and SLL populations, highlighting their clonal unrelatedness, and suggested that strategies such as standardised chemotherapy and interleukin‐4 inhibition could address chemo‐resistance, offering an alternative to bone marrow transplantation. These cell‐type‐specific insights from DVP can guide personalised oncological treatments [64].

#### 3.2.2 Coupling MALDI‐MSI With Immunohistochemistry

Recently, Bindi et al. [65] explored the potential of a newly developed technique called MALDI‐HiPLEX‐IHC, which combines the multiplexing power of MSI with immunohistochemistry. This method involves staining tissues with a range of antibodies linked to photocleavable mass tags, enabling highly multiplexed, targeted imaging of biomolecules within tissue. In a proof‐of‐concept study, the authors demonstrated a workflow that sequentially integrates MALDI‐HiPLEX‐IHC with untargeted spatial proteomics on a single FFPE tissue section, using clinical clear cell renal cell carcinoma as a model. This approach allowed the stratification of histologically similar tumour cores of the same grade, based on differences in lymphocyte populations, particularly T regulatory cells. Furthermore, when combined with untargeted spatial proteomics, the study revealed proteomic alterations linked to these lymphocyte infiltration patterns. These findings highlight the potential of this integrated workflow to map and characterise the molecular environment of tumour‐infiltrating lymphocytes, providing valuable insights into the molecular impact of immune cells within the tumour microenvironment [65].

#### 3.2.3 Algorithms for Multi‐Omics Data Integration

With the emergence of the ‘spatial multi‐omics’ trend, a key challenge lies in integrating different omics datasets, not only on the spatial level but also bioinformatically. Data generated from various techniques or instruments often come in different file formats and require distinct software and algorithms for analysis. Traditional image fusion has been practised for over a decade, which involves co‐registering histology and immunohistochemistry images with low spatial resolution MALDI‐MSI dataset using mathematical models [66]. This process relied on imaging sharpening and model prediction algorithms to correlate regions of interest in microscopic images and ion intensity maps from MALDI‐MSI, typically through supervised and labour‐intensive analysis [66]. As we are advancing towards single‐cell resolution, multi‐modal algorithms are evolving to accommodate this scale [67] and application on multi‐omics analyses.

Recent advancements in 2024 have highlighted the integration of metabolomic MSI with spatial transcriptomic analyses, as demonstrated by Vicari et al. [68] This approach uses an interactive application that utilises histology staining and analyte intensity mapping to correlate spatial and molecular data effectively. Further advancing the field, Schwenzfier et al. [69] introduced the Fluorescence Integrated Single‐Cell Analysis Script (FISCAS) that automatically generates single‐cell mass spectra with optimised morphometric parameters, enhancing the detection of cellular heterogeneity by incorporating fluorescence staining of samples prior to MALDI‐MSI acquisition [69]. FISCAS facilitates automated selection of tightly defined measurement regions, thereby minimising the acquisition of off‐target pixels. In addition, it leverages established algorithms for cell segmentation and co‐registration, facilitating the rapid compilation of single‐cell spectra. By enabling more precise measurements at sub‐cellular resolution, FISCAS significantly enhances the potential for downstream multi‐modal data integration, making it a valuable tool for advanced analytical workflows.

### 3.3 MS Instrumentation, Acquisition and Data Analysis

In the recent years, the MS instrumentation for MALDI‐MSI had breakthroughs in both spatial resolution and sensitivity, as well as the ability to map fragment ions at MS/MS level. The limitations of MALDI‐MSI, such as the lack of MS/MS data and dimensions for accurate identification, have been gradually addressed through several technologies: (1) Ion mobility MS, which adds an another level of separation by collision cross‐section (CCS) value based on ion conformation, (2) iprm‐PASEF that enables parallel mapping of fragment ions at the MS/MS level and (3) MALDI‐2, a post‐ionisation technique, enhances ionisation efficiency. Furthermore, data analysis algorithms have also been improved via integration of deep learning to precision mapping of analytes at single‐cell level.

#### 3.3.1 Ion Mobility

**FIGURE 4:**
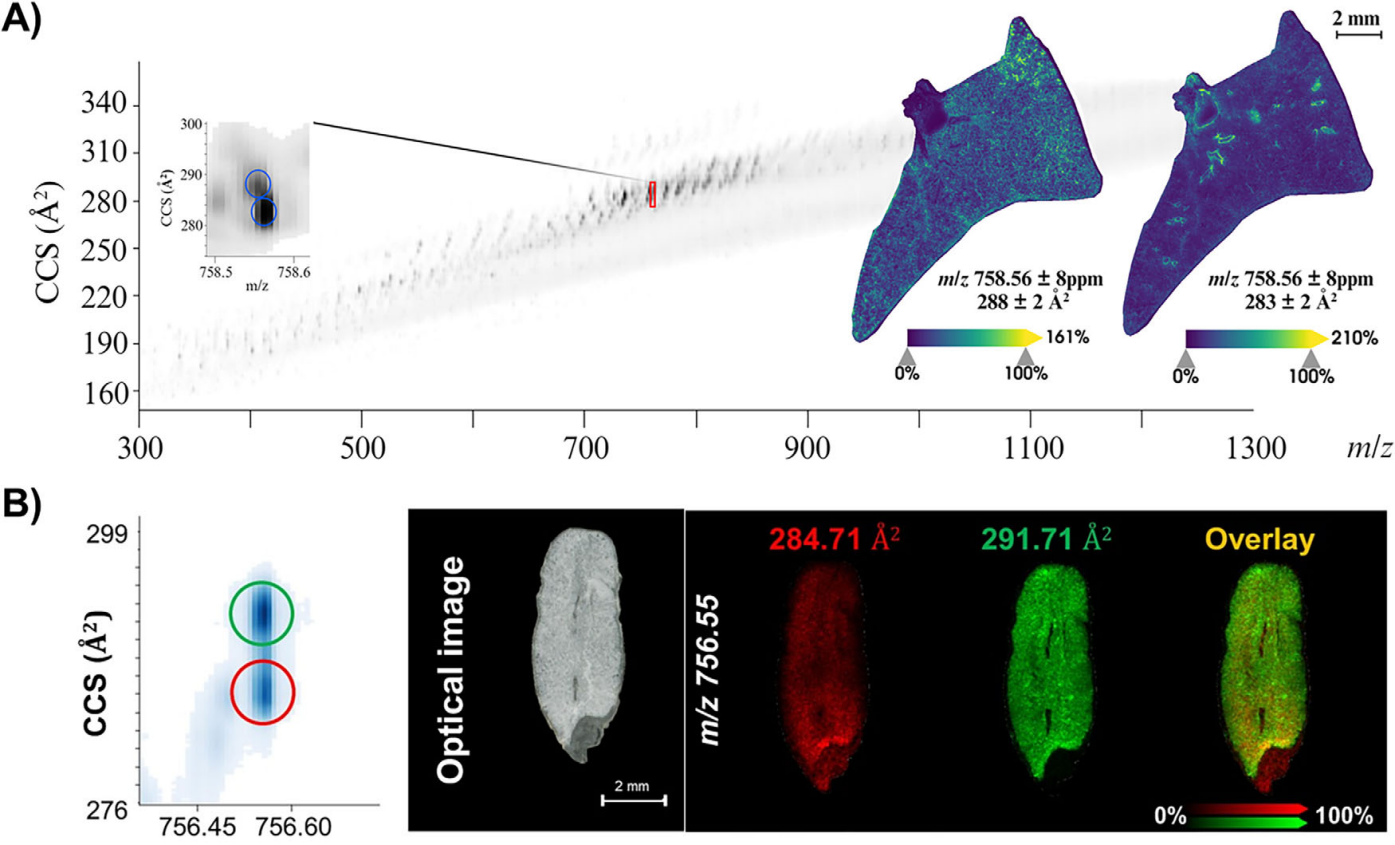
MALDI‐MSI coupled with trapped ion mobility separation (TIMS) enabled differentiation of isobaric molecules with distinct distribution patterns. (A) Identification of isobaric species (*m*/*z* 758.56) with different collision cross section (CCS) values in mouse lung tissue. Ion distribution image revealed distinct localisation patterns of isobaric species. Adapted from Ngai et al. [12] (fig. 3) with permission from John Wiley and Sons. (B) Similar observation was made in oat grain tissue for *m*/*z* 756.55, with one species distributed throughout the grain (red), while the other localised in the white endosperm region (green). Adapted and modified from Lau et al. [81] (fig. 6) with permission from Elsevier.

Ion mobility (*K*) is measured and reported as inverse reduced ion mobility (1/*K*_0_) mobility, which is determined by the CCS value, based on the precursor ion's shape in the gas phase. In the recent years, trapped ion mobility mass spectrometry (TIMS) technology has made an advancement to allow an extra dimension of separation of ions to allow precise identification of analytes, allowing the separation of isobaric and isomeric analytes (Figure 4) [70]. This technology has been widely used for LC‐MS/MS proteomics [71, 72]. The dual TIMS cell system of the timsTOF fleX (Bruker Daltonics, Germany) instrument enables the parallel accumulation serial fragmentation (PASEF) acquisition algorithm by synchronising ion storage and release using ion trap devices. This is followed by quadrupole‐based ion selection and fragmentation to maximise ion usage and enhance sensitivity for fragment ion detection [73]. Utilising the latest algorithm, known as iprm‐PASEF (Bruker Daltonics, Germany), Li et al. has demonstrated an improvement of peptide identification in MALDI‐MSI by selecting up to five precursor ions with predefined 1/*K*_0_ and *m*/*z* range, as supported by successful Mascot search results, without compromising sensitivity and spectrum quality [73]. The distribution of fragment ions can then be visualised using the latest version of SCiLS Lab (Bruker Daltonics, Germany). While iprm‐PASEF can only be performed on five precursors, this is still a significant step forward from conventional MALDI‐MSI experiments that only provide MS1 information.

#### 3.3.2 MALDI‐2

Oversampling has been a technique to increase spatial resolution by moving the stage in increments smaller than the laser beam. With only one part of the laser beam used for ionisation, a smaller spot size is generated [74]. However, a compromise in sensitivity may occur [75]. To overcome this, MALDI‐2 laser post‐ionisation technique, demonstrating double amounts of analytes being detected per pixel, based on the timsTOF fleX MALDI‐2 product brochure (Bruker Daltonics, Germany). Stage positioning accuracy plays an important part in this technique, as minimum step size and reproducibility are critical to avoid stripping and other artifacts [76]. MicroGRID by Bruker Daltonics (Bruker Daltonics, Germany) can address this to allow single cell and subcellular MSI experiment for precise stage movement. McKinnon et al. has also demonstrated post‐ionisation technique using Orbitrap Elite mass spectrometer (Thermo Fisher Scientific GmbH, Germany) coupled to an intermediate pressure MALDI/ESI source (Spectroglyph LLC, USA) with a Nd:YAG laser (Nano L‐DPSS) (Litron Lasers, UK) as MALDI‐2, resulting in a high spatial resolution of 6 µm pixel size [76].

### 3.4 Machine Learning and Deep Learning

MALDI‐MSI analyses and interpretations of complex molecular data can now be enhanced with the integration of machine learning (ML) and deep learning (DL) techniques. ML models trained using MALDI‐MSI data have demonstrated accurate diagnostic results. For instance, a study on cutaneous squamous cell carcinoma utilised supervised ML to achieve 92.3% predictive accuracy, with pathologist validation exceeding 99% [77]. Similarly, a study on basal cell carcinoma achieved 99% classification accuracy, with metabolome profiling identifying 189 significant signals as potential tumour markers [78]. These examples exemplify the potential of ML‐enhanced MALDI‐MSI in clinical disease diagnosis.

Several platforms have integrated ML and DL into MALDI‐MSI data analysis workflows in 2024. For example, METASPACE‐ML, a successor to the existing METASPACE platform for metabolite annotations, was introduced. This ML model was trained on an extensive animal and plant datasets, and it was shown to outperform the conventional rule‐based approach, providing higher precision at low false discovery rate (FDR) thresholds and greater capability to detect trace metabolites [79]. Furthermore, MEISTER, which utilises deep learning techniques to enhance data processing and throughput for tissue and single‐cell analysis, was developed. This advanced MS framework incorporates innovations such as: (i) a deep‐learning signal reconstruction method to improve mass resolution, (ii) a multimodal image registration technique for 3D tissue reconstructions and subsequent quantitative chemical analysis and (iii) a computational approach to integrate cell‐specific chemical profiles with tissue imaging data [80]. With MEISTER, researchers have built cell‐type‐specific chemical libraries from more than 13,000 single cells in different rat brain regions. They were then mapped to tissue images, revealing heterogeneity in lipid distribution at tissue and single‐cell levels [80]. This technique represents a milestone in the integration of spatial omics for multiscale biochemical characterisation.

## 4 Summary and Outlook

Over the years, MALDI‐MSI transitioned from a novel molecular imaging technique used for biomedical research to an established tool with ever‐growing applications. Here, we described the diverse range of MALDI‐MSI applications in studying different types of organisms. While MALDI‐MSI continues to play a prominent role in disease and drug development studies, we foresee the growth of MALDI‐MSI applications into even more diverse fields. For example, the growing debate around per‐ and polyfluoroalkyl substances (PFAS) pollution have intrigued researchers to investigate the accumulation of microplastics in mammalian or plant tissues. MALDI‐MSI has also demonstrated biodiversity and nature preservation purposes, with the ability to characterise metabolic interactions in ecosystems. MALDI‐MSI may also be a valuable technique in food and cosmetic industries, especially for allergen testing.

Significant advancements in MALDI‐MSI were witnessed in 2024, particularly in sample preparation techniques, instrumentations to enhance spatial resolution and ion mobility separation, integration of spatial multi‐omics and the expansion into single‐cell analysis. The integration of artificial intelligence may also drive MALDI‐MSI forward, improving data analysis, automating workflows and becoming more user‐friendly. With its continuing evolution into a multi‐disciplinary tool, the future of MALDI‐MSI looks promising.

## Author Contributions

**Yuen Tung Ngai**: conceptualisation, visualisation, writing – original draft preparation, writing – review and editing. **Darren Lau**: conceptualisation, visualisation, writing – original draft preparation, writing – review and editing. **Parul Mittal**: conceptualisation, supervision, writing – review and editing. **Peter Hoffmann**: conceptualisation, funding acquisition, supervision, writing – review and editing.

## Conflicts of Interest

The authors declare no conflicts of interest.

## Data Availability

The authors have nothing to report.
